# Scaffolds from medical grade chitosan: A good choice for 3D cultivation of mesenchymal stem cells

**DOI:** 10.55730/1300-0152.2633

**Published:** 2022-09-19

**Authors:** Merve ÇAPKIN YURTSEVER, Özge Ekin AKDERE, Menemşe GÜMÜŞDERELİOĞLU

**Affiliations:** 1 Department of Bioengineering, Adana Alparslan Türkeş Science and Technology University, Adana, Turkey; 2 Department of Bioengineering, Hacettepe University, Ankara, Turkey; 3 Department of Chemical Engineering, Hacettepe University, Ankara, Turkey; 4 Department of Nanomedicine and Nanotechnology, Hacettepe University, Ankara, Turkey

**Keywords:** Medical grade chitosan, genipin, stem cell, scaffold

## Abstract

Chitosan has high biocompatibility, supports proliferation of many cells, and can be a good carrier for various growth factors. However, low attachment ratio and spheroid formation of several stem cell types on plain chitosan scaffolds/films is still a problem. In this study, it was aimed to obtain 3D scaffolds using medical grade chitosan (MC) with a high deacetylation degree (DD ≥ 92.6%) to overcome the spheroid formation of rat adipose tissue derived mesenchymal stem cells (rAdMSCs) on control chitosan (C, DD = 75%–85%) scaffolds. Genipin was used as a biological chemical crosslinker, and glycerol phosphate salt was used both as a pH adjusting agent and physical crosslinker. MTT and SEM analyses and live/dead staining indicated the increase in the attachment, cell viability, and proliferation of rAdMSCs on MC scaffolds with or without crosslinking when compared to the cells in spheroid formation on control scaffolds. Moreover, filamentous actin protein organization of rAdMSCs was found to be triggered on the crosslinked MC scaffolds. In conclusion, plain medical grade chitosan scaffolds with or without crosslinking prevented spheroid formation, supported the attachment, proliferation, and organization of rAdMSCs indicating that medical grade type of chitosan scaffolds with high DD can be a very good candidate as 3D carriers in stem cell cultivation.

## 1. Introduction

Chitosan is one of the most promising biopolymers with its high biocompatibility, low cost, high modifiability, biodegradability, antifungal, antibacterial and antihemorrhagic properties. There are numerous studies that investigate the biocompatibility of chitosan with different cell types (Muzzarelli et al., 2015; Jeong et al., 2017; Pandey et al., 2017; Ahmed et al., 2018). Recently, the studies including chitosan and stem cells with their precious proliferation and differentiation capacities (Busilacchi et al., 2013; Muzzarelli et al., 2016) become prominent in regenerative medicine. Mesenchymal stem cells (MSCs) are considered as an ideal cell source in tissue engineering owing to their ability of self-renewal and multi-lineage differentiation. They are isolated from various tissues, including umbilical cord, bone marrow, and adipose tissue. Among these tissues, adipose tissue represents an abundant and easily accessible source for MSCs. Therefore, adipose tissue derived MSCs (AdMSCs) have a considerable amount of applications in tissue engineering studies (Akdere et al., 2019; Lotfi et al., 2019) but it is still an important issue to provide a suitable microenvironment which is a key factor regulating cell fate (Santos et al., 2012).

Spheroid formation of some types of stem cells on plain chitosan scaffolds is one of the most important challenges from the point of tissue engineering. In our previous study, it was shown that spheroid formation of human dental pulp stem cells (hDPSCs) on the chitosan (75%–85% DD) scaffolds was prevented by fibronectin immobilization (Asghari Sana et al., 2017). In another study, spontaneous spheroid formation of human AdMSCs was shown on the chitosan films and it was indicated that their proliferation was inhibited while they were in spheroid form. However, these spheroid forming cells were able to adhere to another cell culture surface and showed better proliferation and differentiation capacities indicating spheroid formation can trigger stemness of AdMSCs (Cheng et al., 2012). Spheroid formation of human AdMSCs was also shown on electrospun chitosan and chitosan/hyaluronan materials and lack of focal adhesion points of the same cells was indicated by vinculin staining (Petrova et al., 2019). In another study, it was shown that poly (Ɛ-caprolactone) (PCL)-chitosan nanofibers induced spheroid formation of MSCs, and these substrates could be more suitable than conventional monolayer culture plates for in vitro MSCs expansion in 3D cultures (Jhala et al., 2020).

It is known that cationic properties of chitosan affect its biocompatibility (He et al., 2015; Kafi et al., 2019). In a study cationic property of water-soluble carboxymethyl chitosan was altered by using crosslinking agent, genipin, in the range of 0.5%–10% (w/v). Increased proliferation of rat bone marrow MSCs was shown in the presence of high genipin concentrations which causes to decrease of free amino groups of chitosan (He et al., 2015). In another study, chitosan and chitosan/collagen scaffolds were obtained by phase-separation following freeze-drying to investigate the behavior of human MSCs. It was shown that proliferation and osteogenic differentiation of human MSCs on pure chitosan or collagen scaffolds was lower than that of the chitosan/collagen (1:1, w/w) scaffolds. These results were attributed to the inadequate adhesion motifs on the chitosan scaffolds and the absence of interconnectivity on the collagen scaffolds (Kafi et al., 2019). In a study, PCL and chitosan blends in different ratios were used to obtain double-layer membranes with different porosities for human bone marrow derived MSCs (hMSCs). Cellular proliferation was quite low on the membranes including high ratio chitosan when compared to the lower ones. In addition, the oxygen uptake rate of hMSCs was decreased by increasing chitosan concentration (Das et al., 2019). In different studies, the relation between Ca^2+^ and MSCs spheroid formation were investigated on chitosan membranes. It was suggested that upregulation of Wnt11 gene expression which can be affected by Ca^2+^ may play a role in the spheroid formation of MSCs (Yeh et al., 2012, 2014). In our previous study, it was reported that a few numbers of AdMSCs attached onto chitosan scaffold and exhibited rounded shape when compared to the hydroxyapatite and boron containing hydroxyapatite coated chitosan scaffolds (Akdere et al., 2019).

The chemical and physical properties of chitosan biopolymer can change according to its source, degree of deacetylation (the ratio of glucosamine to *N-*acetylglucosamine units is described as DD), and methylation. Medical grade chitosan is a type of biopolymer which is produced under a controlled production area and in compliance with ISO 13485 and ISO 22442. Research studies investigating the behavior of stem cells on medical grade chitosan are rare. On the other hand, medical grade chitosan biopolymer was chosen for some biomaterial studies such as preparation of nerve conduits (Stenberg et al., 2016; Ronchi et al., 2018), protein releasing nanoparticles (Stie et al., 2019) and hydrogels for bone tissue regeneration (Bush et al., 2016).

In this study, attachment and proliferation behavior of rat AdMSCs were investigated both on plain chitosan scaffolds with or without medical grade properties for the first time. It was aimed to prevent the spheroid formation of rat AdMSCs on medical grade chitosan scaffolds with or without crosslinking. The scaffolds were obtained by the combination of sol-gel transition and freeze-drying methods. Medical grade chitosan with high DD (≥ 92.6%) was used to increase stem cell attachment and proliferation at different polymer ratios (2% and 3% w/v). Genipin, as a natural crosslinker with low toxic properties, was used as a covalent crosslinker and glycerol phosphate was used both as a pH adjusting agent and physical crosslinker. Nonmedical grade chitosan scaffolds with lower DD (75%–85%) were used as a control group.

## 2. Materials and methods

### 2.1. Preparation of scaffolds with or without crosslinking

Medical grade chitosan (MC) was used to prepare scaffolds (extracted from snow crab, degree of deacetylation ≥92.6%, Heppe, Germany) with or without crosslinking. Chitosan solutions in 2% and 3% (w/v) concentrations were prepared in 0.2 M acetic acid in ultra-pure water. Half of the chitosan solutions were poured into 24-well plates and directly frozen at −20 °C, while the other half was crosslinked in the presence of 0.12 M glycerol phosphate (in ultra-pure water, Sigma, Germany) and 1mM genipin (in absolute ethanol, Challenge Bioproducts, Thailand). Solutions were poured into 24-well plates (2 mL/well) and gelation were triggered at 37 °C for 2 h in a closed environment to prevent drying of the gels. After gelation, the scaffolds were frozen at −20 °C for 2 days. Then, all scaffolds with or without crosslinking were transferred into freeze-dryer (Christ, Germany) at −80 °C and lyophilized for 4 days. After complete drying, they were taken out from the wells and soaked in 96% (v/v) ethanol for stabilization of the scaffolds (Gültan et al., 2021). Excess ethanol was witdrawn, the samples were frozen at −20 °C for 2 days and then, they were transferred into freeze-dryer at −80 °C and lyophilized for 4 days, again. Lyophilized scaffolds were cut into disk form with 9 mm diameter and 2 mm thickness.

Two types of scaffolds were obtained using medical grade chitosan at different ratios (w/v) without crosslinking: MC2 (2%) and MC3 (3%). In addition, crosslinked medical grade chitosan scaffolds, MCG2 and MCG3, were also obtained at the same chitosan ratios. Control chitosan scaffolds (C2, 2%, w/v) were prepared using medium molecular weight chitosan (extracted from seashells skeleton of animals, 75%–85% deacetylation degree, Sigma, Germany, ) as in our previous study (Seda Tiğli et al., 2007).

### 2.2. Characterization of scaffolds

The porous structure of chitosan scaffolds was examined by a scanning electron microscope (SEM, JEOL JSM-5200) at 15.00 kV after gold-palladium coating. SEM images were used to determine the average pore sizes of the scaffolds by Image J software (NIH, Bethesda, MD). The difference in the functional groups of scaffolds was investigated by ATR-FTIR (Attenuated Total Reflectance–Fourier Transform Infrared Spectroscopy, Thermo Scientific FTIR-SMART ITR, Diamond ATR) before and after crosslinking. To determine the water sorption capacities (Q) of dried chitosan scaffolds they were immersed into phosphate buffer saline (PBS, pH 7.4) at 37 °C and weighed after removing excess PBS at defined time intervals (W_s_) and at dry state (W_d_). Then, Q = (W_s_–W_d_)/W_d_ formula was used for calculation. Micro-CT analysis of scaffolds was performed using the Skyscan1272 microtomography (Bruker). The scan files were reconstructed using a modified Feldkamp algorithm as provided by Skyscan. The effect of the different chitosan ratios and genipin crosslinking on scaffold porosity was investigated using the Bruker CTAn program.

### 2.3. Culture of rat AdMSCs on the chitosan scaffolds

Cell culture studies were carried out with rat adipose tissue derived mesenchymal stem cells (rAdMSCs) at passages between 4 and 6. These primary cells were isolated and characterized by three different lineage differentiation and CD marker analysis in our previous study under the approval of the Non-invasive Clinical Research Ethic Committee of Hacettepe University (number: 2015/32-04, Ankara, Turkey) (Akdere et al., 2019). The cells were cultured in 1% (v/v) L-glutamine (Sigma, Germany), 15% (v/v) FBS (fetal bovine serum, Biowest, France) and antibiotics (10 units/mL penicillin, 10 μg/mL streptomycin, 50 μg/mL gentamycin, 250 ng/mL Amphotericin B; Sigma, Germany) containing alpha-Minimum Essential Medium (α-MEM, Sigma, Germany), cell maintenance medium (CMM).

The scaffolds with 9 mm diameter and 2 mm thickness were transferred into 70% (v/v) ethanol solution for sterilization (2h) before cell seeding. Then, they were washed with sterile PBS three times and put under UV-C light for second sterilization for 2 h. The surfaces of 24-well tissue culture plates were covered by presterilized 2% agar (w/v in ultra-pure water) to prevent cell adhesion. Sterilized scaffolds were transferred into agar covered wells and immersed in CMM for conditioning overnight. The day after, the conditioning medium was aspirated, and the cells were seeded onto the scaffolds in 2×10^5^ cells/50 μL concentration. Twenty μL of CMM was added onto the cell-seeded scaffolds in every 2 h to prevent drying. One mL medium was added onto the cell-seeded scaffolds after 6 h, at the end of cell attachment period. This period is needed for the complete attachment of rAdMSCs onto the scaffolds. The cells were cultured at 37 °C in 5% CO_2_ atmosphere for 18 days.

### 2.4. Cell morphology by SEM and confocal microscopy

The morphology of rAdMSCs on the scaffolds was investigated by SEM (JEOL JSM-5200) analysis on days 7 and 18. In brief, the scaffolds were fixed with 2.5% (v/v) glutaraldehyde (in PBS), washed with PBS twice, and dehydrated immersing into ethanol series (30, 50, 70, 90, and 100%). At the last step, hexamethyldisilazane was applied to the samples for complete drying and the samples were coated with gold/palladium before SEM analysis (Asghari Sana et al., 2017). To investigate the cytoskeleton of rAdMSCs on the scaffolds the cells were immunostained using Alexa Fluor^®^ 488 conjugated anti F-actin antibody (1:100 dilution, Invitrogen, USA, Ex:488 and Em:528) which stains actin filaments and 10 μg/mL propidium iodide which stains cell nucleus (PI, Sigma, Germany, Ex:543 and Em:605). In brief, at the end of the cultivation scaffolds were washed with PBS and fixed in 2.5% (v/v in PBS) glutaraldehyde solution for 20 min at room temperature. Then, they were washed with PBS three times and permeabilized in 0.1% Triton X-100 (v/v in PBS) for 5 min. After washing three times with PBS, the samples were incubated in anti-F-actin antibody and PI containing PBS/A (1% w/v, bovine serum albumin including PBS) for 30 min at room temperature, in dark. At the end of incubation, all the samples were washed with PBS/A for three times and investigated under the confocal microscope (Zeiss LSM 510).

### 2.5. Cell proliferation by MTT assay

Proliferation of rAdMSCs was assessed by MTT (3-(4,5-dimethylthiazol-2-yl)-2,5 diphenyltetrazolium bromide, Sigma, Germany) assay on days 8, 13, and 18. The culture medium was aspirated from the scaffolds and 600 μL 0.25 mg/mL MTT reagent including maintenance medium without FBS was added onto the samples and incubated at 37 °C in 5% CO_2_ incubator for 3h. After the incubation period, the supernatant was aspirated from the samples and formazan crystals were dissolved in isopropanol solution (with 0.04 M HCl). Corresponding absorbance values were recorded by a microplate reader Biochrom, Asys UVM 340 at 570 nm with reference to 690 nm.

### 2.6. Cell viability by live/dead staining

The viability of rAdMSCs on the scaffolds was visualized by live/dead staining. Live cells break down Calcein-AM (Sigma, Germany, Ex:488 and Em:528), which is a cell membrane permeable molecule, into calcein molecule with green fluorescence. Dead cells are stained by Ethidium homodimer-1 (Eth-1, Sigma, Germany, Ex:543 and Em:605) in red indicating disintegrated cell membrane. Scaffolds which were taken from cell culture medium were incubated in 1 μM Calcein-AM, 1 μM Eth-1, and 1% (v/v) L-glutamine including PBS/A for 30 min at room temperature in dark. After incubation, all samples were washed in 1% (v/v) L-glutamine containing PBS/A once and analyzed under the confocal microscope (Zeiss LSM 510) in the same solution as soon as possible.

### 2.7. Statistical analysis

Statistical differences among the groups (at least n = 3) were evaluated by GraphPad Prism 8 software. One-way ANOVA and Tukey-Kramer post hoc tests were used to determine the significant differences among the groups.

## 3. Results

### 3.1. Characteristics of scaffolds

**Figure 1:**
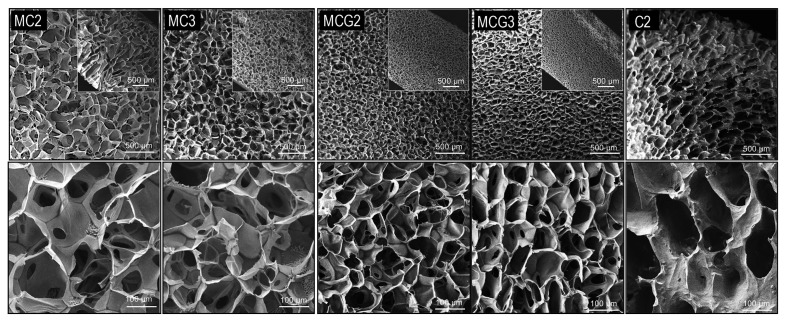
SEM images of the scaffolds. Embedded small images show cross-section of the scaffolds. MC2 and MC3 are scaffolds prepared with 2% and 3% chitosan ratios without crosslinking, respectively. MCG2 and MCG3 are scaffolds prepared with 2% and 3% chitosan ratios with crosslinking, respectively. Control chitosan, C2, prepared using 2% (w/v) low DD chitosan solution.

**Figure 2:**
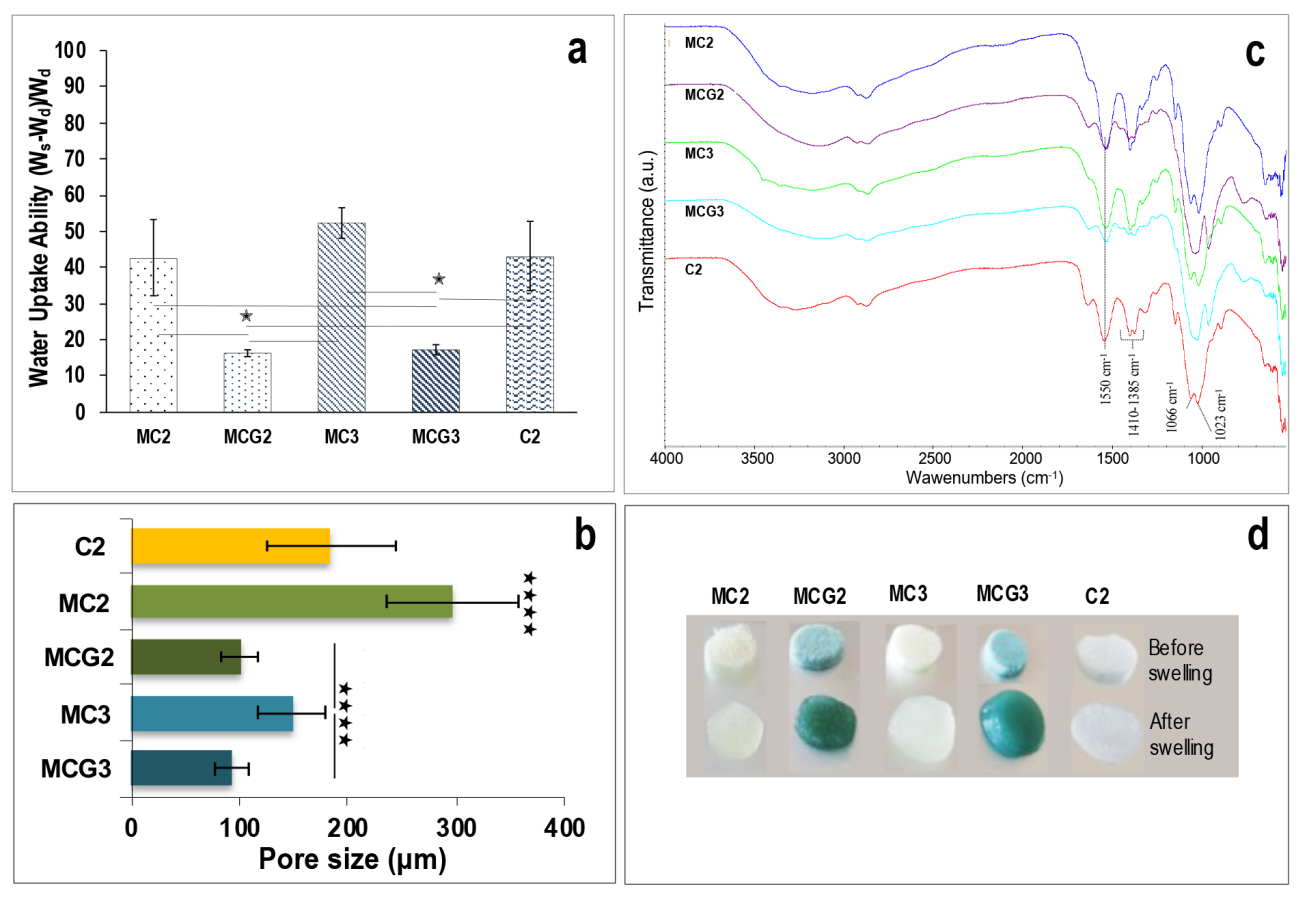
a) Water uptake abilities, W_d_ is the weight of dried hydrogel and W_s_ is the weight of swollen hydrogel, b) pore sizes, c) ATR-FTIR spectra and d) macroscopic images of the scaffolds. Chitosan ratios (w/v) of the scaffolds without crosslinking: MC2 (2%), MC3 (3%); with crosslinking at the same chitosan ratios MCG2 and MCG3. Control chitosan, C2, prepared using 2% (w/v) low DD chitosan solution (p < 0.05 *; p < 0.01 **; p < 0.001; *** p < 0.0001 ****).

Properties of scaffolds are given in Table. Micro-CT analysis indicated that uncrosslinked MC2 (porosity: 83.95%) and MC3 (porosity: 85.54%) scaffolds have the most porous structure when compared to the crosslinked groups. Genipin crosslinking decreased porosity in MCG2 (porosity: 79.47%) and MCG3 (porosity: 62.90%) groups. Control chitosan (porosity: 87.38%) scaffold (C2) showed similar porosity when compared to MC2 and MC3 groups. Since closed porosities for all the scaffolds were so slight, it can be concluded that the entire pore structure of the scaffolds was interconnected. Surface morphologies and pore structures of the scaffolds were also investigated by SEM analysis. As seen in the surface and cross-section views of the scaffolds, pore distribution was homogeneous for MC2 and MC3 groups and the pores inside the samples were interconnected. C2 scaffolds showed homogeneously and some elongated pore structures. Crosslinking did not affect the interconnectivity of the scaffolds (Figure 1). However, pore sizes of crosslinked scaffolds (MCG2 100 ± 16 mm; MCG3 93 ± 16 mm) decreased significantly (p < 0.00001) in both groups when compared to the groups without crosslinking (MC2 196 ± 60 mm; MC3 148 ± 32 mm) as seen in Figure 2a. The pore size of C2 scaffolds was similar (184 ± 60 mm) as MC3 scaffolds.

Crosslinking affected water uptake abilities of the scaffolds as seen in Figure 2b. The water uptake ratio of the crosslinked scaffolds decreased more than 50% when compared to the uncrosslinked scaffolds (p < 0.05) as due to the decreased pore sizes in the crosslinked scaffolds. ATR-FTIR analysis indicated that intensities of amide II peaks, bending vibrations of NH_2_, at 1550 cm^−1^ were decreased after crosslinking (Figure 2c). This decrease can be an indicator of the covalent binding of NH_2_ groups of chitosan by genipin. Characteristic peaks of chitosan molecule between 1410 cm^−1^, 1385 cm^−1^; C-O stretching and CH bending (Melo et al., 2014; Skwarczynska et al., 2018) were decreased after crosslinking. In addition, the strong band at 1066 cm^−1^ and 1023 cm^−1^ corresponds to C-O stretching. Macroscopic images of scaffolds before and after swelling in PBS are given in Figure 2d. Genipin crosslinked scaffolds turn green after 2 h incubation at 37 °C.

### 3.2. Cell culture studies

**Figure 3:**
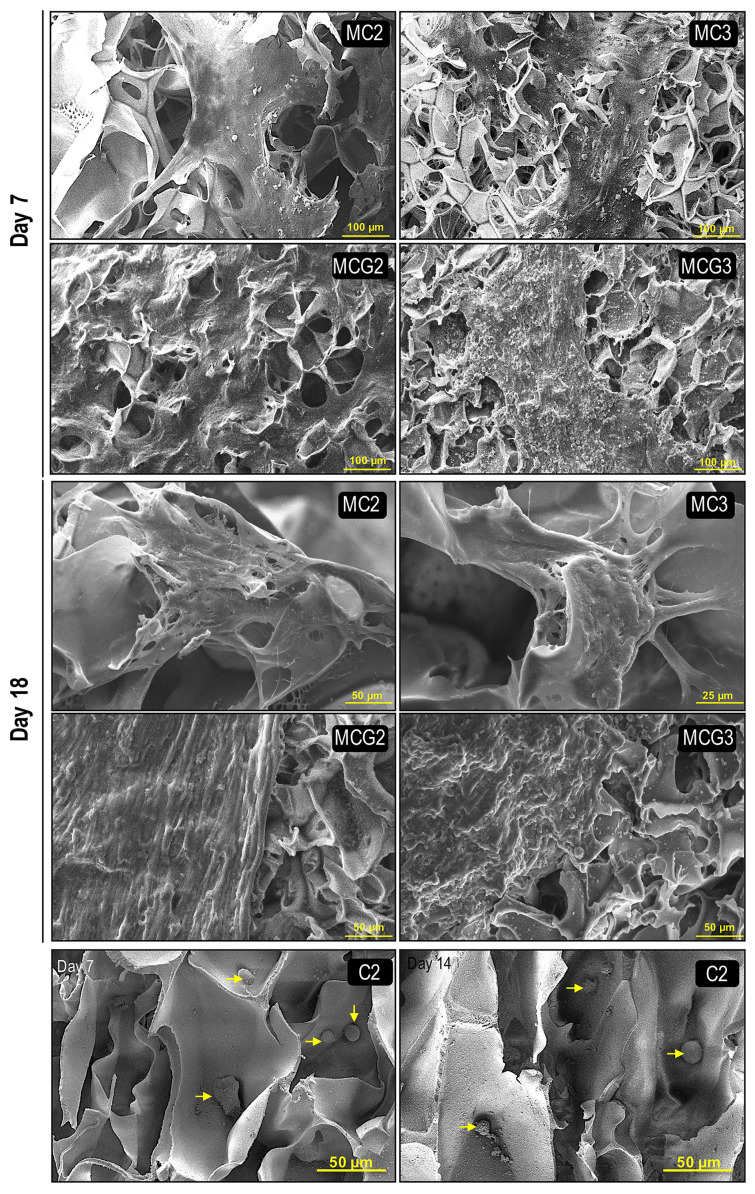
SEM images of rAdMSCs cultured on the scaffolds at days 7 and 18. Chitosan ratios (w/v) of the scaffolds without crosslinking: MC2 (2%), MC3 (3%); with crosslinking at the same chitosan ratios MCG2 and MCG3. Control chitosan, C2, prepared using 2% (w/v) low DD chitosan solution.

Morphologies of rAdMSCs were determined by SEM analysis on days 7 and 18 (Figure 3). The medical grade chitosan scaffolds supported attachment and proliferation of rAdMSCs when compared to the control group (C2). The spheroid formation of rAdMSCs was distinctly prevented on medical grade chitosan scaffolds when compared to the C2 scaffolds. The cells were localized and well spread on MC2 and MC3 scaffolds. The number of attached and spread cells increased on the surface of the genipin crosslinked MCG2 and MCG3 scaffolds. However, the cells were not able to migrate through the pores of MCG2 and MCG3 scaffolds because the sizes of the pores might be small for rAdMSCs. The increased cell density on MCG2 and MCG3 scaffolds was clearly seen on day 18 with the evidence of coverage of the scaffold surfaces by the cells (Figure 3).

**Figure 4:**
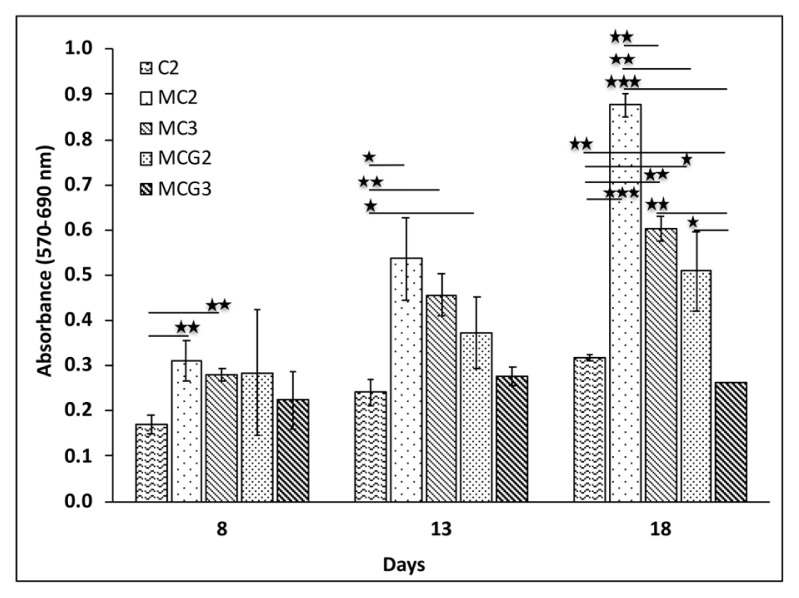
Viabilities of rAdMSCs on the scaffolds by MTT assay on days 8, 13, and 18. Chitosan ratios (w/v) of the scaffolds without crosslinking: MC2 (2%), MC3 (3%); with crosslinking at the same chitosan ratios MCG2 and MCG3. Control chitosan, C2, prepared using 2% (w/v) low DD chitosan solution (p < 0.05 *; p < 0.01 **; p < 0.001 ***).

Viabilities of rAdMSCs on the scaffolds were investigated by MTT analysis (Figure 4). Cell proliferation was better in the medical grade chitosan groups when compared to the C2 group on days 8 and 13. On the other hand, cell viability was significantly higher in MC2 and MC3 groups (p < 0.001) when compared to the C2 group on day 8. Cellular viability increased in the upcoming days on all scaffolds except MCG3 and there were significantly much more cells in MC2 group when compared to the other groups (p < 0.0001 and p < 0.001). Rat AdMSCs were able to triple their number on MC2 scaffolds on day 18 when compared to the cell viability on day 8.

**Figure 5:**
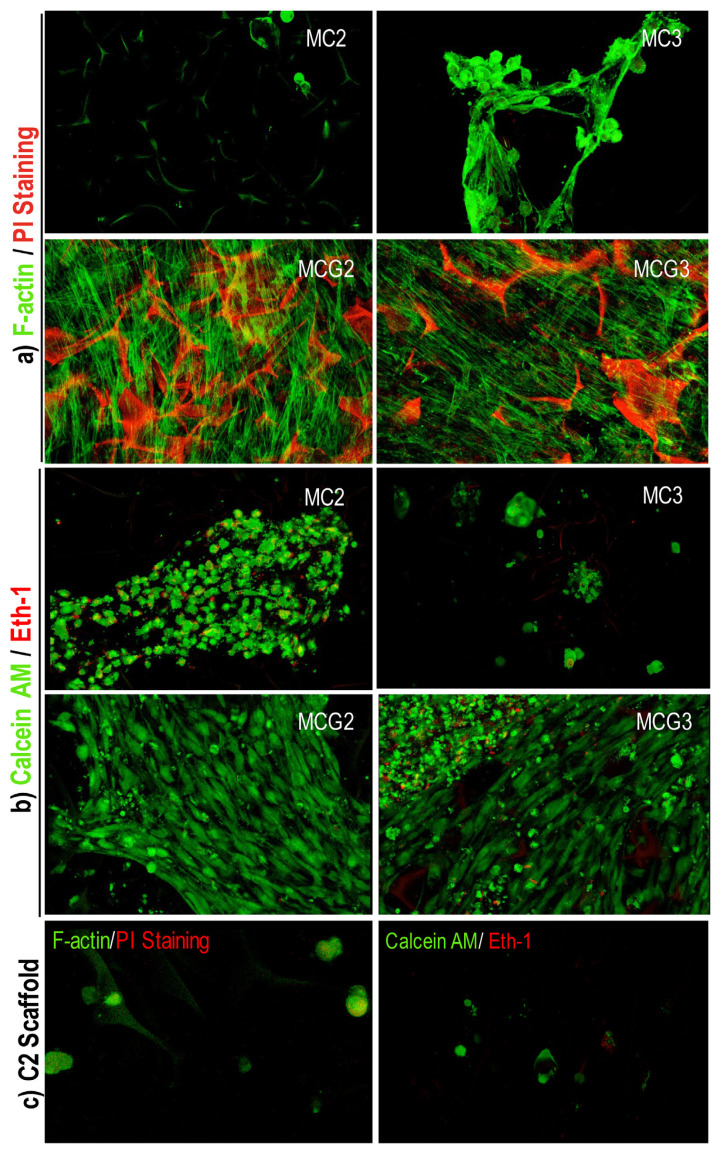
Confocal images of rAdMSCs cultured on the scaffolds at day 18 (20x). Actin filaments were stained using anti F-actin antibody in green and the nucleus was stained using propidium iodide (PI) in red. Calcein AM shows esterase activity in live cells (green) and ethidium homodimer-1 (Eth-1) stains the nucleus of damaged cells (red). Chitosan ratios (w/v) of the scaffolds without crosslinking: MC2 (2%), MC3 (3%); with crosslinking at the same chitosan ratios MCG2 and MCG3. Control chitosan, C2, prepared using 2% (w/v) low DD chitosan solution.

**Figure 6:**
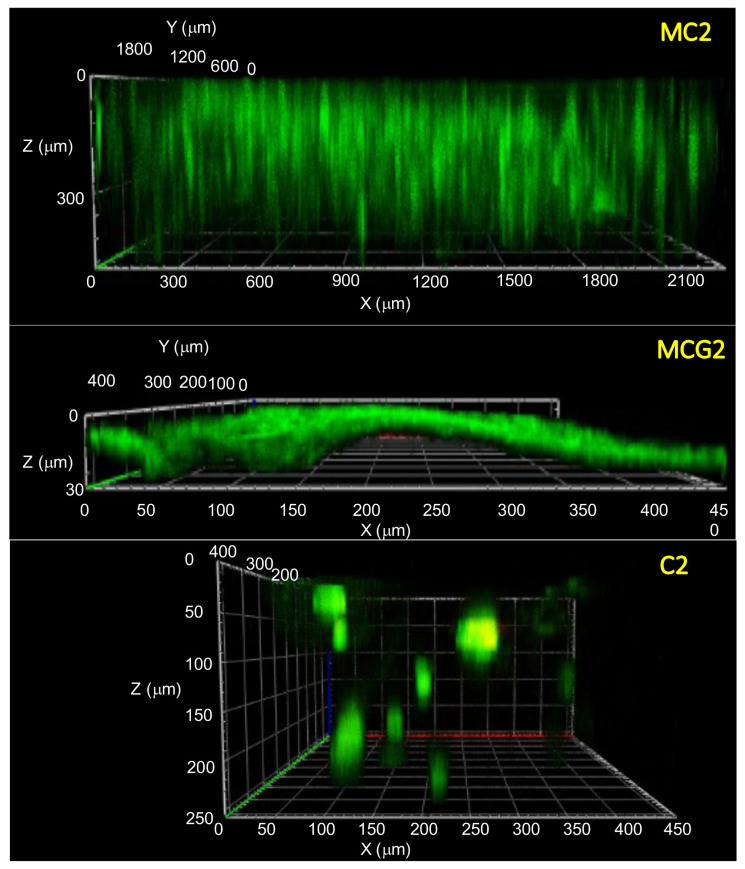
3D confocal images of rAdMSCs cultured on the scaffolds at day 18 (MC2 4x; MCG2 and C2, 20x). Actin filaments were stained using anti F-actin antibody in green. Chitosan ratios (w/v) of the scaffolds without crosslinking: MC2 (2%), with crosslinking at the same chitosan ratios MCG2. Control chitosan, C2, prepared using 2% (w/v) low DD chitosan solution.

The cytoskeleton of rAdMSCs was determined by staining of fibrous actin proteins on day 18 (Figure 5). The organization of fibrous actin fibers was clearly seen in the cells cultured on MCG2 and MCG3 scaffolds. However, the cells showed thinner and elongated morphology on MC2 and MC3 scaffolds with undistinct actin fiber organization as seen in Figure 5a. Lower fluorescence intensities of the spread cells showed their ability to migrate through the pores of MC2 scaffolds. The chitosan structure tends to be stained with PI, and this behavior is more pronounced in the genipin-crosslinked chitosan scaffolds as seen in Figure 5a. Therefore, it was difficult to observe the structure of nuclei. Live/dead staining was performed to show cellular viability on the scaffolds. There were more viable cells on MCG2 and MCG3 scaffolds (Figure 5b). Dead cells were seen on the scaffolds with red color where the cells were spread and proliferated in big clusters. Mesenchymal stem cells including rAdMSCs generally tend to die if they proliferate in restricted areas such as clumps. Due to cluster formation, diffusion of nutrients, oxygen, and waste through clumps is limited in a size-dependent manner (Cesarz and Tamama, 2016). There were some dead cells on MCG3 scaffolds in the cell clusters. F-actin/PI and live/dead staining images of rAdMSCs on C2 scaffolds are given in Figure 5c. As it was clearly seen from the images, the cells were not able to attach and spread on the surface of C2 scaffolds and they showed spherical morphology. The cross-section view of 3D confocal images for F-actin staining are given in Figure 6. MC2 scaffolds significantly supported cell migration through the pores, however, the cells formed thick cell layer on the top of the MCG2 scaffolds. In addition, spheroid formation of AdMSCs both on the surface and inside the pores of C2 scaffolds were shown by green clusters.

## 4. Discussion

In the current study, we investigated the adhesion and proliferation behavior of rAdMSCs on the different types of chitosan scaffolds. The spheroid formation of rAdMSCs was prevented on the medical grade chitosan scaffolds with higher DD (^3^92.6%) in the presence or absence of crosslinking agents when compared to the control chitosan scaffolds with lower DD (75%–85%). In addition, the spreading and proliferation of rAdMSCs were also supported by these scaffolds. These results may be related to the purity of medical grade chitosan, the difference between DDs, and the presence of genipin crosslinking agent in the structure. Purification of chitosan from impurities such as residual heavy metals which may cause an increased high chronic toxicity is necessary to be used as a scaffold material. In a study, several commercial chitosan specimens were analyzed and medical grade chitosan is found to be purest in terms of the content of heavy metals (Bezrodnykh et al., 2020). Properties of chitosan scaffolds such as stiffness, crystallinity, and rate of degradation can be affected by the DD. Thus, a variety of the surface properties of chitosan related to DD plays a crucial role in cell behaviors such as migration, proliferation, and differentiation (Hsu et al., 2004; Chou et al., 2016). Tığlı et al. and Reys et al. observed that high DD chitosan scaffolds supported cell proliferation more than low DD chitosan scaffolds (Tığlı et al., 2007; Reys et al., 2017). Also, chitosan with low DD was found to induce an acute inflammatory response as a result of the fast rate of degradation, while chitosan with high DD caused minimal inflammation (Kurita et al., 2000). Chou et al. obtained cell spheroids from corneal keratocytes on different chitosan substrates. They investigated that DD-mediated surface roughness/stiffness of chitosan coatings affected migration, proliferation, and differentiation of keratocytes. Therefore, chitosan with low DD could lead to augmenting cell migration and spheroid formation (Chou et al., 2016).

Many studies have indicated that the spheroid formation of MSCs on several biomaterials or using the hanging drop technique preserves their stemness properties (Baraniak et al., 2012; Hsu et al., 2014). This spheroid formation of MSCs has benefits to get large-scale expansion for clinical applications such as cell therapy (Zhang et al., 2015). However, they can easily detach from the surface of the biomaterials, therefore it is not possible to use them for tissue engineering studies in combination with scaffolds (Asghari Sana et al., 2017). In our previous study, the attachment, spreading, and proliferation capacity of human dental pulp stem cells (hDPSCs) were evaluated on the chitosan (75%–85% DD) scaffolds produced by freeze-drying (Asghari Sana et al., 2017). The results showed that hDPSCs on chitosan scaffolds formed spheroids while fibronectin-immobilized chitosan scaffolds strongly supported cellular attachment and proliferation. To enhance the mechanical and bioactive properties of chitosan scaffolds, Akdere et al. coated the scaffolds with HAp (hydroxyapatite) or B-HAp (boron doped hydroxyapatite). The results showed that a high number of AdMSCs attached and proliferated on HAp and B-HAp coated scaffolds when compared to bare chitosan scaffolds (Akdere et al., 2019). In another study, it was reported that MSCs formed spheroids that kept attached for 10 days on the chitosan (77.7% DD) and chitosan-hyaluronan membranes. Also, these membranes helped to maintain the expression of stemness marker genes of MSCs (Huang et al., 2011). Yan et al. demonstrated that periodontal ligament cell (PDL) spheroids formed on chitosan films. Although their self-renewal gene expression was significantly higher in comparison with cells cultured in a monolayer, they exhibited decreased proliferation after spheroid formation compared with monolayer cell culture (Yan et al., 2018). Here, plain medical grade chitosan scaffolds with or without genipin crosslinking supported rAdMSCs attachment and proliferation.

Chitosan can be crosslinked using traditional reagents such as glutaraldehyde, tripolyphosphate, ethylene glycol, and diisocyanate to modify its properties. However, these synthetic crosslinking reagents can be toxic to the cells. Genipin is a biological crosslinker that is extracted from *gardenia jasminoides* fruits with its low toxicity. It reacts with NH_2_ groups of chitosan promptly thus producing blue/green-colored hydrogels. Besides the resulting crosslinked complexes are not cytotoxic, they have some beneficial effects such as antiinflammatory and antioxidant properties due to the genipin in their structure (Muzzarelli, 2009). As seen in Figure 1, pore structure and interconnectivity of MCG2 and MCG3 scaffolds were not affected by genipin crosslinking. However, micro-CT analysis showed that pore sizes were different for scaffolds with or without genipin crosslinking. Pore size is an important parameter for tissue engineering. Cells cannot migrate towards the center of scaffolds when pores are too small. On the contrary, the surface area is limited if pores are too large. It is stated that the optimal pore size is necessary for successful tissue engineering studies. For instance, studies in bone tissue engineering have indicated a range of mean pore sizes up to 800 μm (Murphy et al., 2010). Also, it was reported that scaffolds with pore sizes ranging from 50 to 200 μm were appropriate for cell infiltration (Dawlee et al., 2005) and they should have more than 80% porosity for nutrient and gas exchange (Ikeda et al., 2014). According to micro-CT results, MCG2, MC3, and C2 scaffolds met this criterion. Pore sizes of the genipin crosslinked scaffolds decreased in both MCG2 and MCG3 groups as seen in Figure 2a. All of the scaffolds had suitable average pore size which was affected by genipin crosslinking and chitosan ratio. Mesenchymal stem cells show heterogeneous cell sizes from 10 to 300 μm for attached cells and they are big when compared to many cell types (Ginzberg et al., 2015). Here, we used rAdMSCs as a stem cell source and in line with our knowledge gained from the studies carried out so far in our laboratory, adipose tissue derived stem cells can show high cell surface area when compared to the bone marrow or dental pulp derived MSCs. Thus, rAdMSCs were able to migrate through MC2 and MC3 scaffolds but tended to form cell sheets on genipin cross-linked MCG2 and MCG3 scaffolds as their pore size decreased. Our results indicated that medical grade chitosan scaffolds, with or without genipin crosslinking, promote the attachment, viability, and proliferation of rAdMSCs by preventing spheroid formation which are prerequisites for tissue engineering studies.

## 5. Conclusions

In conclusion, increased attachment and proliferation of rAdMSCs were observed on the scaffolds prepared by using medical grade chitosan with high DD and with or without genipin crosslinking. Improved physical and chemical properties of our scaffolds are suitable for MSCs, thus the cells do not attach to each other to form cell spheroids. Moreover, our scaffolds may be a preferable candidate which prevents the spheroid formation of MSCs with their enhanced biocompatibility. Overall, these scaffolds that provide a promotive microenvironment for cell attachment, have the potential to be a suitable niche for the regeneration of several tissues by being modified with differentiation agents.

## Availability of data and material

All data generated or analyzed during this study are included in this manuscript (and its supplementary information files).

## Figures and Tables

**Table:** Properties of the chitosan scaffolds.

Scaffold	Chitosan (% w/v)	Genipin (mM)	Glycerol phosphate (M)	Degree of deacetylation (%)	Pore sizes (mm)	Open porosity (%)	Closed porosity (%)	Total porosity (%)
C2	2	-	-	75–85	184 ± 60	87.38	0.00	87.38
MC2	2	-	-	≥92.6	196 ± 60	83.95	0.01	83.96
MCG2	2	1	0.12	≥92.6	100 ± 16	79.47	0.01	79.48
MC3	3	-	-	≥92.6	148 ± 32	85.54	0.01	85.55
MCG3	3	1	0.12	≥92.6	93 ± 16	62.90	0.00	62.90

Chitosan ratios (w/v) of the scaffolds without crosslinking: MC2 (2%), MC3 (3%); with crosslinking at the same chitosan ratios MCG2 and MCG3. Control chitosan, C2, prepared using 2% (w/v) low DD chitosan solution.
